# Supplementary material for: Metabolic and Transcriptional Stress Memory in Sorbus pohuashanensis Suspension Cells Induced by Yeast Extract
Source: Cells. 2022 Nov 24;11(23):3757. doi: 10.3390/cells11233757 (PMC9739749; doi:10.3390/cells11233757)
Supplement: Supplementary file 1 [file cells-11-03757-s001.zip › cells-1980630-supplementary/Supplements-Figure S Final.pdf]

Supplements

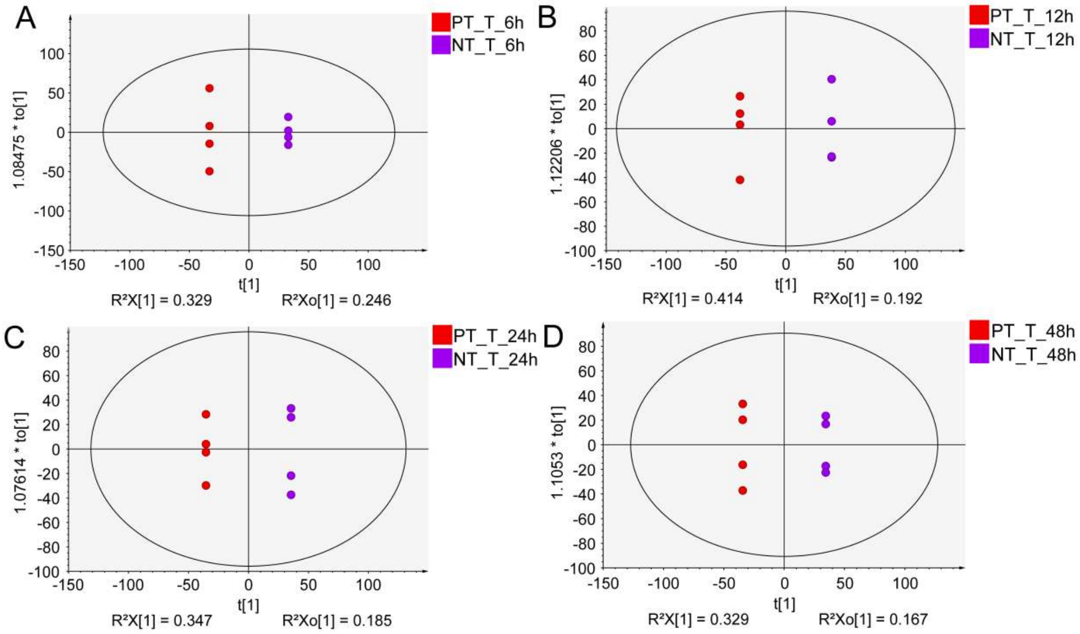

Figure S1 OPLS-DA score plots of the metabolomics of comparing PT_T group and NT_T group at 6 (A), 12(B), 24(C) and 48 (D) HAT.

Supplements

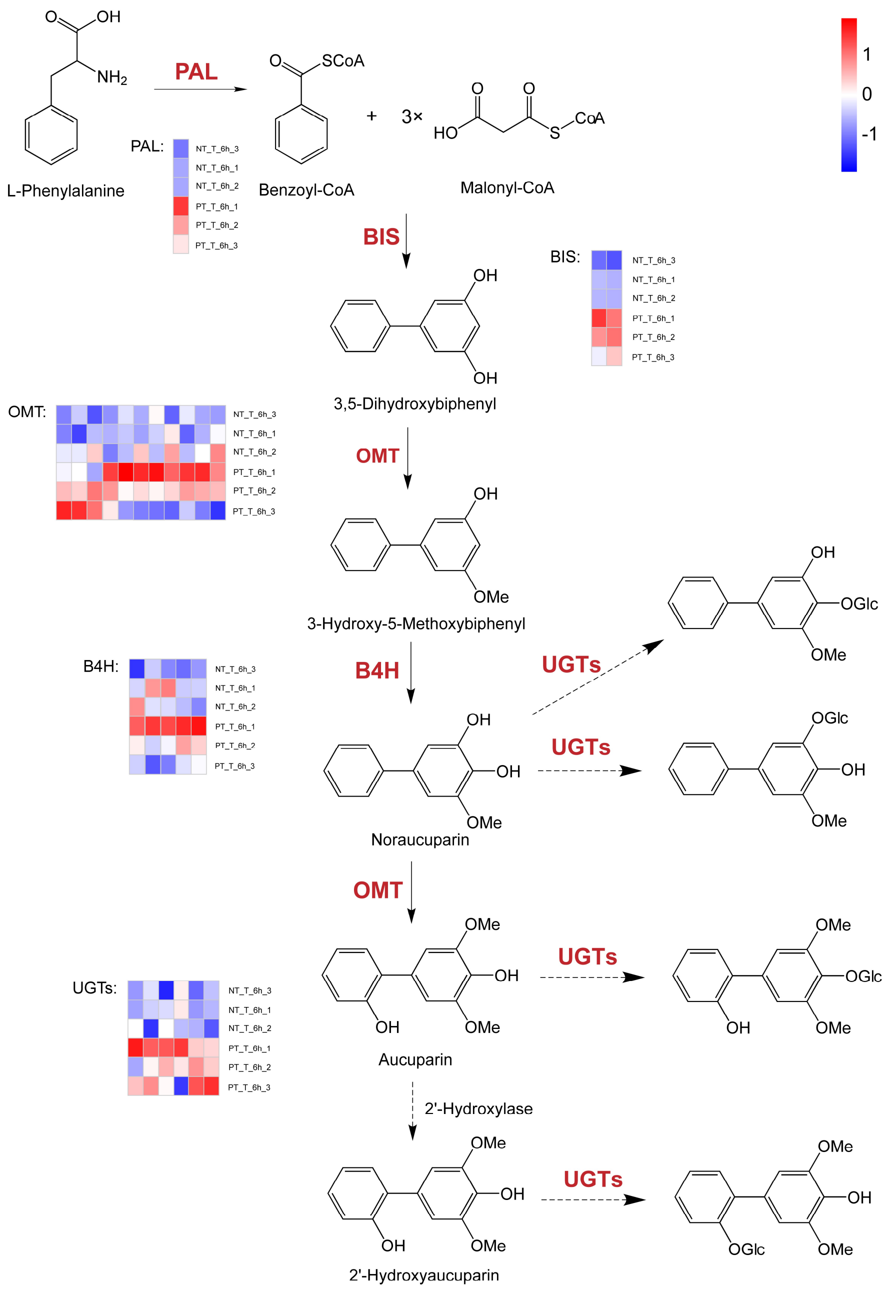

Figure S2 Comparative transcriptomic analysis of structural genes involved in the biphenyl biosynthesis pathway
